# Is the association between sleep and socio-emotional development mediated by weight in toddlers aged 12 to 36 months?

**DOI:** 10.3389/fpsyg.2023.1190081

**Published:** 2023-11-30

**Authors:** Ana Duarte, Silvana Martins, Luís Lopes, Maria José Silva, Cláudia Augusto, Rute Santos, Rafaela Rosário

**Affiliations:** ^1^The Health Sciences Research Unit: Nursing (UICISA: E), School of Nursing of Coimbra (ESEnfC), Coimbra, Portugal; ^2^Research Centre on Child Studies (CIEC), Institution of Education, University of Minho, Braga, Portugal; ^3^Research Centre in Physical Activity, Health and Leisure, Faculty of Sports, University of Porto, Porto, Portugal; ^4^Nursing Research Centre, School of Nursing, University of Minho, Braga, Portugal; ^5^School of Nursing, University of Minho, Braga, Portugal; ^6^Institute of Education, University of Minho, Braga, Portugal

**Keywords:** child development, adiposity, sleep, socio-emotional skills, children

## Abstract

**Introduction:**

Childhood is an important stage for socio-emotional development. Understanding the associations of lifestyle habits with the healthy development of social and emotional skills is crucial for better interventions early in life. This study aims to analyze the association between sleep and socio-emotional development in toddlers aged 12 to 36 months and examine whether weight mediated these associations.

**Methods:**

This study is part of a cluster randomized controlled trial developed in Portuguese childcare centers. A sample of 344 children (176 females) enrolled in the study. Participants’ anthropometrics were measured while at childcare centers using standardized procedures. Body mass index (BMI) was computed as the body weight/height2 (kg/m2) ratio. Sleep quality was collected with the Tayside Children’s Sleep Questionnaire, a 10-item scale that evaluates the child’s ability to initiate and maintain sleep. Two additional questions regarding sleep duration were added. Parental questionnaires assessed the child’s sex and date of birth, socioeconomic status, and total energy intake (TEI). Motor (fine and gross) was assessed using Bayley-III scales and socio-emotional (SE) by the Greenspan Social–Emotional Growth Chart questionnaire. Linear regression models were used to examine the associations between sleep (duration and quality) and SE with adjustments for sex, age, BMI, mothers’ education, motor development, and TEI. Mediation analysis was conducted using path analysis.

**Results:**

SE development was significantly associated with nighttime sleep duration even when adjusted for confounders (β = 0.223; 95% CI: 0.001, 0.004 and β = 0.168; 0.0003, 0.003; respectively). Sleep quality was not significantly associated with SE development, and the weight did not explain the associations between sleep and SE development.

**Conclusion:**

This study supports that sleep duration is directly associated with SE development in toddlers. From a public health perspective, sleep duration should be prioritized in intervention programs to improve socio-emotional development early in life.

## 1 Introduction

The first three years of life are a critical period of rapid growth, during which cognitive, social, emotional, and many other skills develop (Brito et al., 2019). Socio-emotional (SE) development is a neurodevelopmental process influenced by many factors, such as social interaction, cultural characteristics, and other environmental factors (Palmer et al., 2018). This process begins with parents, before conception, and during pregnancy, and matures throughout childhood (Palmer et al., 2018). The development of socio-emotional skills is a gradual process that involves exploration, experience, and interaction with others (e.g., affect sharing, social play), the communication and management of emotions, and the development of relationships (Palmer et al., 2018; Brito et al., 2019). The prevalence of socio-emotional delays appears to be higher in toddlers than in older children (Gilliam, 2005); however, few studies have explored socio-emotional development features in these first years and the variables that could influence them (Vaughn et al., 2015).

Emotion regulation is essential to children’s healthy growth and development. Children who experience some type of adversity, such as maltreatment, poverty, or social risk factors, have greater difficulties in emotion regulation, which seems to have important implications for health behaviors, and ultimately for body mass index (BMI) (Doom et al., 2023). It is possible that some health behaviors, such as the increased overeating, might be a mechanism for coping with negative moods (Doom et al., 2023). Additionally, poorer emotion regulation seems to predict emotional overeating (Gianini et al., 2013), which in turn might contribute to overweight and obesity. In contrast, BMI also interferes with socio-emotional development. According to Henninger and Luzes (Henninger and Luze, 2010), both being underweight and overweight in early childhood can result in many consequences for cognitive, motor, and socio-emotional outcomes (Henninger and Luze, 2010; Faught et al., 2017; Albataineh et al., 2019). Some studies indicate that sleep duration and quality are associated with important health outcomes like physical and mental development or emotional regulation (Vaughn et al., 2015; Chaput et al., 2016; Deng et al., 2021). School-age children who sleep less or have sleep problems are more likely to have more emotional delays, relational problems, and other social challenges when compared to those who sleep more or have a better quality of sleep (Touchette et al., 2007; Vaughn et al., 2015). Furthermore, at these ages, shorter sleep duration and sleep problems have been associated with difficulties during daytime activities, including school performance (Touchette et al., 2007; Vaughn et al., 2015).

Although epidemiological studies suggest that short sleep duration is a problem during childhood (Cappuccio et al., 2008; Matricciani et al., 2012; Ogilvie and Patel, 2017), research on toddlerhood is still limited (Bayley, 2006; Vaughn et al., 2015). Additionally, there is a lack of evidence on the associations between sleep and socio-emotional development in toddlers (Hoyniak et al., 2020). Furthermore, the role of adiposity on socio-emotional development during this age group is overlooked, and per our understanding, only one study found an inverse association between these variables (Must and Strauss, 1999).

While some studies report a direct effect of sleep on socioemotional development, it is possible a mediating effect of adiposity on these sleep-induced socioemotional alterations, likely to be explained by inflammatory processes (affects social experiences) that can be associated with social disconnection and in altering sensitivity to social world.

Despite the associations between sleep (particularly, sleep deprivation) with inflammation and adiposity as a path of socioemotional development delay, the interrelations between sleep, adiposity and socioemotional development need further clarifications, particularly at younger ages (Eisenberger and Moieni, 2020). The total energy intake assumes a crucial role in the examination of the relationship between sleep, adiposity and socioemotional development. It can function as a proxy for variables such as body size, sleep, and metabolic efficiency in the analysis of nutrient-disease connections (McCullough and Byrd, 2022). Also, motor development is recognized as a trigger of various developments that extend motor behavior, including perception and cognition, language and communication, emotional expression and regulation, physical growth, and overall health, among others (Adolph and Hoch, 2020).

Therefore, this study aims to examine the associations between sleep and socio-emotional development in toddlers aged 12 to 36 months and investigate whether adiposity mediated these associations.

## 2 Materials and methods

### 2.1 Study design and participants

This study is part of a cluster randomized controlled trial developed in Portuguese childcare centers. A minimum of 20 children in each childcare center was required to participate in the study, with no other inclusion criteria. Any disability that prevented children from being assessed while at the childcare center was deemed as an exclusion criterion. A total of 344 children, 168 boys (48.8%) and 176 girls (51.2%), belonging to 15 childcare centers and whose parents consented to participate in the study. The mean age of children was 23.6 (6.3) months. At the moment of evaluation, each child was asked about their participation and provided oral assent.

### 2.2 Socioeconomic status

Mothers’ education was obtained through a sociodemographic questionnaire (e.g., what is the mother’s highest level of education?). The answers were further recorded below than higher education, and higher education, and more.

### 2.3 Anthropometrics

Anthropometric measures were performed by trained researchers who measured children’s length and weight while at childcare centers. Recumbent length (12–24 months) was measured with the child lying down using an infant stadiometer placed on a flat and stable surface. If the child could stand and refused to lie down, we measured the standing height and added 0.7 cm to convert it into length (WHO, 2008). Weight was measured with a pediatric scale (SECA 354) and recorded to the nearest 100 g. Measurements were taken with no shoes and while wearing light clothing. Waist circumference was measured to the nearest 0.1 cm, with the child standing, at the umbilicus level.

BMI was computed as the ratio of body weight/height2 (kg/m2), and each child was classified according to the age- and sex-specific BMI (BMI for age) and BMI standard *z*-scores using the WHO Anthro-plus software.^1^

### 2.4 Sleep duration

Sleep duration was collected with the Tayside Children’s Sleep Questionnaire, a 10-item scale that evaluates the child’s ability to initiate and maintain sleep. This questionnaire is validated for children aged 1 to 5 years (Mcgreavey et al., 2005). The items address initial settling, nighttime disruption, and early morning arousal. The first nine questions were summed (the total score - range of 0 to 36 - indicates the severity of the problem, with higher scores indicating greater severity. It is acceptable that a score of 8 or superior suggests sleep problems). The tenth question is to ascertain parents’ perception of a potential night problem and was excluded from data analysis, as considered in previous studies (Mcgreavey et al., 2005).

The wake-up and bedtime during weekdays were reported by parents. Daytime sleep duration (e.g., naps) was reported by childcare professionals during three weekdays, and the meantime was obtained. In the current study, nighttime sleep duration was defined as the bedtime/wake-up time without naps. Total sleep duration included nighttime and daytime sleep.

Cronbach’s alpha of the original questionnaire was 0.85, indicating good internal consistency (Mcgreavey et al., 2005). In the current study, a Cronbach’s alpha of 0.76 was found. The value of Mcdonalds’ omega was also calculated, which was 0.77.

### 2.5 Socio-emotional development

SE development was assessed with the Bayley Scales of Infant and Toddler Development – Third Edition (Bayley-III) (Bayley, 2006), which is adapted from the Greenspan Social–Emotional Growth Chart (Greenspan, 2004). This is a comprehensive scale completed by the child’s parent or other caregiver, designed to discern and assess the child’s emotional competencies such as self-regulation and interest in the world, the ability to communicate needs, the establishment of relationships, the deliberate use of emotions, and the utilization of emotional cues to solve problems (Bayley, 2006).

Bayley-III is considered the most widely used test of general neurodevelopment and is designed for children aged between 1 to 42 months (Brito et al., 2019). The socioemotional scale identifies six stages (with substages), with milestones according to the child’s age, and measures behaviors associated with major milestones in functional and emotional development (Bayley, 2006).

The Cronbach’s alpha of the questionnaire from the original study is 0.90, which indicates a strong internal consistency (Bayley, 2006). For this work, percentiles values were used.

### 2.6 Motor development (fine and gross motor)

Motor development was assessed with Bayley Scales of Infant and Toddler Development — Third Edition (Bayley-III). Bayley-III Gross Motor Scale evaluates motor abilities such as sitting, standing up, and walking, while the Fine Motor Scale assesses fine motor control abilities. These scales were used by trained professionals through direct observation of the child, and it was scored following the procedures described in the manual, according to the child’s age at the starting point (Bayley, 2006). Percentiles of motor development were used in the analysis.

### 2.7 Total energy intake

The child’s eating habits were collected using the two-day food record from two non-consecutive weekdays, completed by the parents and childcare teachers (European Food Safety Authority, 2009). Energy and nutritional intake were estimated using an adapted Portuguese version of the nutritional analysis software ESHA’s Food Processor Plus (ESHA Research Inc., Salem, OR, United States).

### 2.8 Ethical considerations

The study was approved by the Ethics Subcommittee for Life and Health Sciences of the University of Minho (CE.CVS 133/2018), and all child’s legal representatives (parents or caregivers) signed the informed consent. At the moment of evaluation, children assent to participate in the procedures.

This cluster randomized controlled trial was registered in the Clinical Trials database/platform (NCT04082247).

### 2.9 Statistical analysis

Descriptive statistics included central tendency measures and dispersion according to the type of variables.

Linear regression models were performed to examine the associations between sleep duration (predictor) and socio-emotional development (outcome), adjusting for demographic factors (e.g., sex, age), anthropometric measures (BMI z-score), socioeconomic status (mothers’ education), total energy intake, and motor development (fine and gross motor). These covariates were selected based on their possible influence on socio-emotional development in the existing literature. Standardized regression coefficients were used to express the beta coefficients of the regression analyses.

Unadjusted multivariable linear regression models were performed to evaluate the associations between anthropometric measures and socio-emotional development. The goal was to identify the most likely variables that could mediate the association between sleep and socio-emotional development. Beta coefficients and a 95% confidence interval were computed. The anthropometric measures used in this study reflect different aspects of body composition and are considered to be well-correlated with adiposity, even in growing children (Must and Strauss, 1999).

Mediation analysis was performed with path analysis. Mediation analysis was used with the purpose of quantifying and investigating the direct as well as indirect routes through which an antecedent variable X (in our study, sleep) conveys its influence on a consequent variable Y (in our study, socioemotional development) by way of one or more intermediary or mediator variables (in our study, adiposity). We chose to employ mediation analysis over moderation analysis, as the latter delves into examining how the impact of variable X on variable Y hinges on the presence of a third variable or a set of variables – an objective that did not align with the scope of our research. Standardized coefficients and confidence intervals were obtained. Models were adjusted for age, sex, mothers’ education, total energy intake, and motor development.

The chi-square test was used to assess the fit of the models. The level of significance was established at 0.05. The data analysis was performed using IBM SPSS, version 27.0, using Process, version 4.1 for the mediation analysis.

## 3 Results

Table 1 presents the participants’ characteristics of the study, including mean age, sleep measures, socio-emotional and motor development, total energy intake, BMI *z*-score, and mothers’ education.

**Table 1:** Characteristics of the participants.

	All *n* = 344	Boys *n* = 168	Girls *n* = 176	*p*
Age (months)	23.6 (6.3)	24.0 (6.5)	23.3 (6.1)	0.352
Tayside total score	15.3 (7.6)	15.3 (7.9)	15.4 (7.2)	0.968
Nighttime sleep duration (hours/day)	10:16 (00:42)	10:15 (00:36)	10:16 (00:48)	0.786
Daytime sleep duration (hours/day)	2:21 (0:29)	2:20 (0:27)	2:22 (0:31)	0.681
Total sleep duration (hours/day)	12:36 (0:49)	12:35 (0:45)	12:37 (0:53)	0.761
Total Energy Intake (kcal/day)	1103.9 (244.8)	1132.4 (229.7)	1077.4 (256.3)	0.101
Socio-emotional development percentile	55.7 (30.0)	54.8 (28.7)	56.6 (31.2)	0.634
Motor development (fine and gross) percentile	45.8 (25.0)	44.0 (25.4)	47.6 (24.5)	0.187
BMI z-score	0.7 (1.0)	0.7 (0.8)	0.7 (1.2)	0.852
	*n* (%)	n (%)	n (%)	
Mothers’ education				0.357
less than higher education	117 (37.4)	54 (34.8)	63 (39.9)	
higher education	196 (62.6)	101 (65.2)	95 (60.1)	

Nighttime sleep on weekdays was directly associated with socio-emotional development in toddlers (95% CI: 0.001, 0.004; *p* = 0.001) even after adjusting for age, sex, total energy intake, BMI, motor development, and mothers’ education (95% CI: 0.0003, 0.003; *p* = 0.019). In the crude model, daytime sleep was inversely associated with socio-emotional development (95% CI: −0.005, 0.001; *p* = 0.008). We found no significant associations between total sleep duration and socio-emotional development (please, see Table 2).

**Table 2:** Associations between sleep duration and socio-emotional development in toddlers.

Socio-emotional development			
	Unadjusted	Adjusted Model^(1)^	*R* ^2^
Nighttime sleep duration	0.223 (0.001; 0.004)*	0.168 (0.0003; 0.003)*	0.167
Daytime sleep duration	−0.173 (−0.005; −0.001)*	−0.126 (−0.004; 0.0001)	0.161
Total sleep duration	0.062 (−0.001; 0.002)	0.047 (−0.001; 0.002)	0.143

(1) Adjusted model for sex, age, mothers’ education, total energy intake, motor development (fine and gross), and BMI *z*-score.

*p ≤ 0.05. All values are ᵦ (95% CI).

Concerning sleep quality, we found no significant associations with socio-emotional development, even though those toddlers with a problem with sleep quality were associated with a lower socio-emotional development [ᵦ –0.034, 95% CI (−13.302; 8.476)] (Table 3).

**Figure 1:**
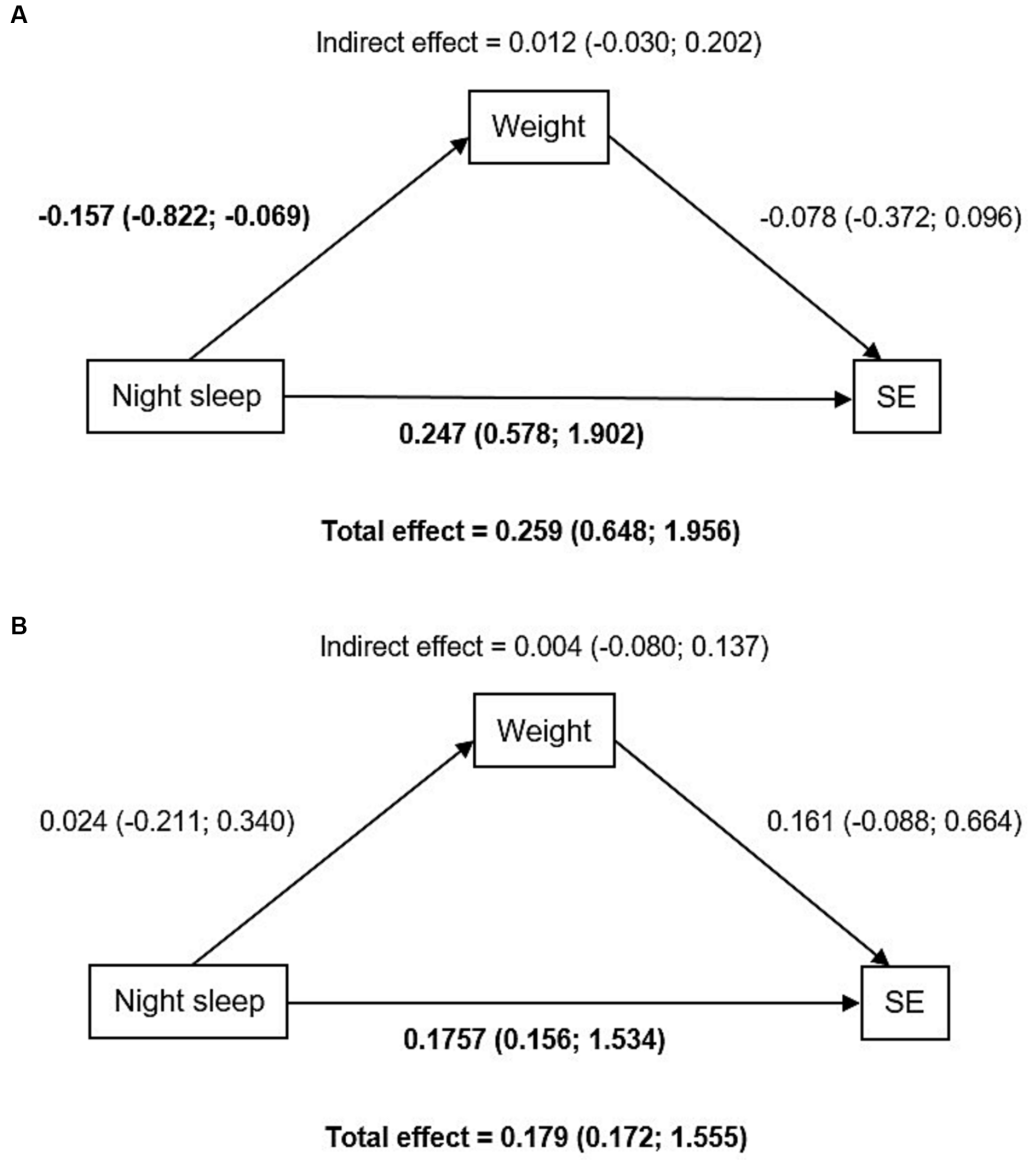
Mediation analysis [β (95% Confidence Interval)]. **(A)** Non-adjusted model. **(B)** Adjusted model for sex, age, mothers’ education, total energy intake (TEI), and motor development (fine and gross motor). Significant results are highlighted in bold.

The crude associations between sleep duration and anthropometric measures were tested, as well as anthropometrics and socio-emotional development (please see Supplementary Tables). Weight was considered a possible mediator. Figure 1 presents the direct effect (nighttime sleep and socio-emotional development) and the indirect effect (mediated by weight). We found a significant direct effect of nighttime sleep on socio-emotional development; however, none of these associations seemed to be mediated by weight, even when adjusting for confounders.

**Table 3:** Associations between sleep quality and socio-emotional development in toddlers.

Socio-emotional development		
	Unadjusted model	Adjusted model^**(1)**^
Quality of sleep (Tayside total score)		
No problem (total score < 8)	Ref.	Ref.
Problem (total score ≥ 8)	−0.083 (−16.281; 4.071)	−0.034 (−13.302; 8.476)

(1) Adjusted model for sex, age, mothers’ education, total energy intake, motor development, and BMI. All values are ᵦ (95% CI).

## 4 Discussion

In the current study, sleep duration during the night is positively associated with socio-emotional development in toddlers aged 12 to 36 months. This association is not mediated by the child’s weight. Our research aligns with previous studies that suggest insufficient sleep, particularly at night, can negatively impact socio-emotional development (Smith et al., 2020). While there has been limited research on this topic (Hoyniak et al., 2020), studies have shown that shorter sleep duration has been associated with poorer socio-emotional skills. For instance, preschoolers who slept for shorter periods had lower social competence and emotional maturity (as reported by their teachers) compared to those who slept for longer periods (Tso et al., 2016). In contrast, longer sleep duration has been positively associated with peer social competence (Vaughn et al., 2015). The first five years of life are particularly crucial in developing strong emotional skills that can benefit children later in infancy and childhood (Williams et al., 2016).

Taveras et al. (2017), with a sample of 1,046 preschool children, found that a sleep time of fewer than 10 h was associated with higher emotional and behavioral difficulties. Insufficient sleep duration at two years has been shown to be prospectively associated with a 32% higher risk of experiencing emotional delay at the age of 3 years (Jansen et al., 2011).

Indeed, sleep plays a critical role in human development, impacting not only learning and memory but also social, emotional, and creative thinking abilities (Jiang, 2020). These effects may be attributed to the different types of sleep, namely non-rapid eye movement (NREM) sleep and rapid eye movement (REM) sleep. During REM sleep, a hyperlimbic and hypoactive dorsolateral prefrontal activation and a normal function of the medial prefrontal cortex promote adequate emotional regulation (Vandekerckhove and Cluydts, 2010; Jiang, 2020). NREM sleep, with the reactivation of the hippocampal-neocortical circuits, also contributes to emotional and cognitive processing (Vandekerckhove and Cluydts, 2010).

The development of socio-emotional skills is influenced by the environment surrounding children, particularly by their parents and other caregivers (Palmer et al., 2018). Research has shown that the lack of emotional support from a nurturing adult can impede emotional regulation in early childhood and potentially affect lifelong health outcomes (Pascoe et al., 2016). On the other hand, a supportive emotional environment is essential for children’s emotional regulation and their ability to cope with adversity throughout their lives (Pascoe et al., 2016).

In the present study, total sleep duration (including nap time) is not significantly associated with socio-emotional development; however, nighttime sleep duration is directly associated with these emotional skills. We were unable to perform analyses that account for different groups of sleep (such as short, normal, and long sleep) due to the limited number of cases in the categories of short and long sleep. Therefore, we are unable to establish associations based on specific sleep duration thresholds.

Nighttime sleep duration has been associated with lower emotional and behavioral difficulties (Zheng et al., 2021), which is in line with our findings. In a sample of children aged 2 to 6 years, predisposed to obesity, Zheng et al. (2021) identified that a 1-h increase in night sleep time was associated with a reduction of 1.02 points in emotional and behavioral difficulties. This association is still stronger after adjusting for sleep problems and lifestyles. Conversely, our findings reveal a direct association between nighttime sleep and socio-emotional development, even in the adjusted model.

Daytime sleep (nap time) is a controversial sleep period. Some studies indicate this sleep time benefits memory performance and cognitive development in children (Horváth et al., 2016; Jiang, 2020); however, these results are not consistent with those from another study that considers wakefulness as a better generalization of word meanings in toddlers with 2.5 years of age (Werchan and Gómez, 2014). While our current study found an association between daytime sleep and lower socio-emotional development, this association was no longer significant when potentially confounding factors were taken into account. Therefore, further research is needed to understand better the relationship between daytime sleep and children’s development.

In the current study, sleep problems are not significantly associated with socio-emotional development, even when adjusted for confounders. This contrasts with previous studies that demonstrated inverse associations between sleep problems (e.g., difficulty or scare of falling asleep, night wakings) and emotional development (Turnbull et al., 2013; Sivertsen et al., 2015; Zheng et al., 2021). A recent study examined data from 570 early adolescents and revealed bidirectional associations between sleep quality and emotional problems such as symptoms of depression and anxiety (Vazsonyi et al., 2022). Furthermore, the analysis of sleep quality profiles in 10,313 individuals (4,913 [47.6%] female) indicated significant associations with longitudinal emotional and behavioral issues from childhood through adolescence (Cooper et al., 2023).

Previous research has found an association between adiposity and socio-emotional difficulties (Chaput et al., 2017), but our current study did not find evidence to support the hypothesis that adiposity mediates the relationship between nighttime sleep and socio-emotional development. It is worth noting that many studies have reported the impact of emotional state on overweight and obesity (Gianini et al., 2013; Shriver et al., 2019; Smith et al., 2020; Doom et al., 2023). Emotional eating is an eating behavior in which individuals turn to food as a coping mechanism for negative emotions. This behavior has been strongly linked to overeating and, as a result, weight gain (Shriver et al., 2019). Emotional eaters tend to consume a greater amount of sweets, salty foods, and other high-energy-dense foods, which can contribute to a positive energy balance and, consequently, overweight or obesity (Shriver et al., 2019; Smith et al., 2020). Poor emotion regulation is a factor that contributes to emotional overeating (Gianini et al., 2013; Doom et al., 2023). We know that toddlers who have better emotion regulation have a lower risk of being overweight or obese compared to those with poor emotion regulation (Miller et al., 2016; Shriver et al., 2019). Furthermore, according to Anderson et al., poorer emotional self-regulation at three years is considered a predictor of obesity at the age of 11 (Anderson et al., 2017).

Another important aspect of emotional overeating in children is the challenge that parents face in managing their children’s behavior, which can lead them to rely on food (often high-calorie foods) as a means of soothing their children. This can create a cycle where children learn to use food as a way to cope with negative emotions, and parents continue to rely on food to manage their children’s behavior (Anzman-Frasca et al., 2012; Smith et al., 2020). This highlights the need for interventions with a family focus, emphasizing that parents develop effective strategies for managing their children’s behavior that does not involve food in order to prevent emotional overeating and promote healthy eating habits in children. Also, community-based interventions that involve family and caregivers should be encouraged to promote long-life achievement in all human development fields (Smith et al., 2020).

We hypothesized that adiposity could affect children’s emotional well-being (Sahoo et al., 2015). While our study did not find a significant mediation effect of weight in the association between night sleep and socio-emotional development, we do acknowledge the relevance of adiposity on children’s development, particularly at the social and emotional levels. While there is evidence linking obesity and socio-emotional difficulties, including lower self-esteem, poorer social functioning, and higher rates of depression and anxiety (Aparicio et al., 2016; Shriver et al., 2019; Valero-García et al., 2021), our study did not find adiposity in the path toward socioemotional development. Therefore, further studies are needed addressing adiposity as a potential factor impacting children’s development and implement interventions that promote healthy weight management.

Childhood is a pivotal stage for development and holds the potential as a valuable period for preventive measures against later socioemotional challenges. Our findings underscore the association between insufficient sleep and poorer socioemotional well-being, emphasizing the importance of fostering healthy sleep habits from an early age. Early intervention to promote improved sleep patterns can contribute to enhanced socioemotional development and, consequently, better overall health. This should be emphasized to parents, involving them and them with the tools necessary to foster healthy habits within their home environments. Additionally, we recognize the significant role of mothers in their children’s behavioral and emotional development. A recent meta-analysis of 193 studies revealed consistent inverse associations between maternal depression and child behavioral and emotional well-being (Goodman et al., 2011).

The majority of mothers participating in this study possess a higher level of education. This aligns with recent findings from the OECD, which indicate that a substantial proportion of Portuguese individuals aged 25 to 34 (47%) have attained a higher level of education (OECD, 2022). While it is conceivable that education is positively linked to various health outcomes, such as obesity and socioemotional development, it is crucial not to underestimate the significance of adequate sleep time.

We agree that our study has several strengths. One of the main strengths is the use of a mediation analysis approach to explore the relationship between sleep, weight, and socio-emotional development in toddlers, which has not been widely studied in this age group. Additionally, we adjusted for major potential confounders in our data analyses, such as mothers’ education, to reduce the likelihood of spurious associations. Another important strength of our study is the training of the researchers involved in the data collection, which helps to ensure the accuracy and consistency of the data. This increases the reliability of our findings about the relationship between sleep, weight, and socio-emotional development in toddlers.

This study also has some limitations. First, the study follows a cross-sectional design with a limited sample size, which limits the generalizability of the findings to other populations. Second, the children’s sleep duration and socio-emotional development were self-reported by parents. It is important to note that the self-report method used in this study may have introduced social desirability bias, where parents may have reported their children’s sleep duration and socio-emotional development in a more positive or socially desirable way than what is actually occurring. This could have led to an underestimation or overestimation of the true association between sleep duration and socio-emotional development. Future studies should consider using more “objective” measures of sleep duration (e.g., actigraphy, polysomnography) and standardized measures of socio-emotional development to reduce the potential for bias in the data.

Sleep plays an important role in children’s development, influencing all-day activities and experiences. Adequate sleep is crucial for children’s physical, cognitive, and emotional development. By prioritizing sleep as a critical aspect of children’s health and development, we can help to ensure that they have the best possible start in life.

Sleep duration is a predictor of socio-emotional development in toddlers; however, this association is not mediated by adiposity. It is important to further explore other predictors of toddlers’ socio-emotional development and the role of sleep duration in this process. In addition, future studies should also examine the potential impact of other lifestyle behaviors, such as physical activity, sedentary behavior, and diet, on children’s development. Interventions aimed at promoting healthy lifestyle behaviors in early childhood may have a positive impact on both physical and socio-emotional development. It is important to invest in early childhood development programs and initiatives that promote healthy behaviors and a supportive environment to help children achieve their full potential.

## Data availability statement

The raw data supporting the conclusions of this article will be made available by the authors, without undue reservation.

## Ethics statement

The studies involving humans were approved by the Ethics Subcommittee for Life and Health Sciences of the University of Minho. The studies were conducted in accordance with the local legislation and institutional requirements. Written informed consent for participation in this study was provided by the participants’ legal guardians/next of kin.

## Author contributions

AD wrote the first draft of the manuscript. AD, SM, CA, MS, LL, and RS contributed to the conception and design of the study. AD and SM organized the database. AD, SM, and RR performed the statistical analysis. SM, LL, and RR wrote sections of the manuscript. All authors contributed to the manuscript revision, read, and approved the submitted version.

## Footnotes

^1^ https://www.who.int./childgrowth/software/en

## References

[ref1] Adolph K. E. Hoch J. E. (2020). The importance of motor skills for development. Nestle Nutr. Inst. Workshop Ser. 95, 136–144. doi: 10.1159/000511511 33166961

[ref2] Albataineh S. R. Badran E. F. Tayyem R. F. (2019). Dietary factors and their association with childhood obesity in the Middle East: a systematic review. Nutr. Health 25, 53–60. doi: 10.1177/0260106018803243 30282516

[ref3] Anderson S. E. Sacker A. Whitaker R. C. Kelly Y. (2017). Self-regulation and household routines at age three and obesity at age eleven: longitudinal analysis of the UK millennium cohort study. Int. J. Obes. 41, 1459–1466. doi: 10.1038/ijo.2017.94, PMID: 28435162 PMC5626576

[ref4] Anzman-Frasca S Stifter CA Birch LL. Temperament and childhood obesity risk: a review of the literature [internet]. (2012). Available at: www.jdbp.org 10.1097/DBP.0b013e31826a119f 23095495

[ref5] Aparicio E. Canals J. Arija V. De Henauw S. Michels N. (2016). The role of emotion regulation in childhood obesity: implications for prevention and treatment. Nutr. Res. Rev. 29, 17–29. doi: 10.1017/S0954422415000153 27045966

[ref6] Bayley N. Bayley scales of infant and toddler development. Texas: United States of America: Psych Corp (2006).

[ref7] Brito N. H. Fifer W. P. Amso D. Barr R. Bell M. A. Calkins S.. (2019). Beyond the Bayley: neurocognitive assessments of development during infancy and toddlerhood. Dev. Neuropsychol. 44, 220–247. doi: 10.1080/87565641.2018.1564310, PMID: 30616391 PMC6399032

[ref8] Cappuccio F. P. Taggart F. M. Kandala N. B. Currie A. Peile E. Stranges S.. (2008). Meta-analysis of short sleep duration and obesity in children and adults. Sleep 31, 619–626. doi: 10.1093/sleep/31.5.619, PMID: 18517032 PMC2398753

[ref9] Chaput J. P. Gray C. E. Poitras V. J. Carson V. Gruber R. Birken C. S.. (2017). Systematic review of the relationships between sleep duration and health indicators in the early years (0-4 years). BMC Public Health 17:855. doi: 10.1186/s12889-017-4850-2, PMID: 29219078 PMC5773910

[ref10] Chaput J. Philippe G. C. E. Poitras V. J. Carson V. Gruber R. Olds T.. (2016). Sleep and health indicators in school-aged children and youth. Appl. Physiol. Nutr. Metab. 41, S266–S282. doi: 10.1139/apnm-2015-0627, PMID: 27306433

[ref11] Cooper R. Di Biase M. A. Bei B. Quach J. Cropley V. (2023). Associations of changes in sleep and emotional and behavioral problems from late childhood to early adolescence. JAMA Psychiatry 80, 585–596. doi: 10.1001/jamapsychiatry.2023.0379, PMID: 37017952 PMC10077137

[ref12] Deng X. He M. He D. Zhu Y. Zhang Z. Niu W. (2021). Sleep duration and obesity in children and adolescents: evidence from an updated and dose–response meta-analysis. Sleep Med. 78, 169–181. doi: 10.1016/j.sleep.2020.12.027, PMID: 33450724

[ref13] Doom J. R. Young E. S. Farrell A. K. Roisman G. I. Simpson J. A. (2023). Behavioral, cognitive, and socioemotional pathways from early childhood adversity to BMI: evidence from two prospective, longitudinal studies. Dev. Psychopathol. 35, 749–765. doi: 10.1017/S0954579421001887, PMID: 35545317 PMC9652481

[ref14] Eisenberger N. I. Moieni M. (2020). Inflammation affects social experience: implications for mental health. World Psych. Off. J. World Psych. Assoc. 19, 109–110. doi: 10.1002/wps.20724, PMID: 31922673 PMC6953558

[ref15] European Food Safety Authority (2009). General principles for the collection of national food consumption data in the view of a pan-European dietary survey. EFSA J. 7:1435. doi: 10.2903/j.efsa.2009.1435

[ref16] Faught E. L. Ekwaru J. P. Gleddie D. Storey K. E. Asbridge M. Veugelers P. J. (2017). The combined impact of diet, physical activity, sleep and screen time on academic achievement: a prospective study of elementary school students in Nova Scotia, Canada. Int. J. Behav. Nutr. Phys. Act. 14:29. doi: 10.1186/s12966-017-0476-0, PMID: 28274260 PMC5343372

[ref17] Gianini L. M. White M. A. Masheb R. M. (2013). Eating pathology, emotion regulation, and emotional overeating in obese adults with binge eating disorder. Eat. Behav. 14, 309–313. doi: 10.1016/j.eatbeh.2013.05.008, PMID: 23910772 PMC4015336

[ref18] Gilliam WS. Prekindergarteners left behind: expulsion rates in state prekindergarten systems. (2005). Available at: https://www.fcd-us.org/wp-content/uploads/2016/04/ExpulsionPolicyBrief.pdf

[ref19] Goodman S. H. Rouse M. H. Connell A. M. Broth M. R. Hall C. M. Heyward D. (2011). Maternal depression and child psychopathology: a meta-analytic review. Clin. Child. Fam. Psychol. Rev. 14, 1–27. doi: 10.1007/s10567-010-0080-1, PMID: 21052833

[ref20] Greenspan S. Greenspan social-emotional growth chart: A screening questionnaire for infants and young children. San Antonio Tex: Psych Corp (2004).

[ref21] Henninger W. R. IV Luze G. J. (2010). Differences in parental perceptions of the socio-emotional development of underweight, overweight, and typically weighted children in a low-income sample. J. Child Health Care 14, 250–260. doi: 10.1177/1367493510370221 20534635

[ref22] Horváth K. Liu S. Plunkett K. (2016). A daytime nap facilitates generalization of word meanings in young toddlers. Sleep 39, 203–207. doi: 10.5665/sleep.5348, PMID: 26237777 PMC4678333

[ref23] Hoyniak C. P. Bates J. E. McQuillan M. E. Staples A. D. Petersen I. T. Rudasill K. M.. (2020). Sleep across early childhood: implications for internalizing and externalizing problems, socioemotional skills, and cognitive and academic abilities in preschool. J. Child Psychol. Psychiatry 61, 1080–1091. doi: 10.1111/jcpp.13225, PMID: 32173864 PMC7812691

[ref24] Jansen P. W. Saridjan N. S. Hofman A. Jaddoe V. W. V. Verhulst F. C. Tiemeier H. (2011). Does disturbed sleeping precede symptoms of anxiety or depression in toddlers? The generation R study. Psychosom. Med. 73, 242–249. doi: 10.1097/PSY.0b013e31820a4abb, PMID: 21257976

[ref25] Jiang F. (2020). Sleep and early brain development. Ann. Nutr. Metab. 75, 44–54. doi: 10.1159/000508055 32564032

[ref26] Matricciani L. Olds T. Petkov J. (2012). In search of lost sleep: secular trends in the sleep time of school-aged children and adolescents. Sleep Med. Rev. 16, 203–211. doi: 10.1016/j.smrv.2011.03.005, PMID: 21612957

[ref27] McCullough L. E. Byrd D. A. (2022). Total energy intake: implications for epidemiologic analyses. Am. J. Epidemiol. 192, 1801–1805. doi: 10.1093/aje/kwac071, PMID: 35419586

[ref28] Mcgreavey J Mcgreavey JA Donnan PT Pagliari HC Sullivan FM. The Tayside children’s sleep questionnaire: a simple tool to evaluate sleep problems in young children. Child Care Health Dev. (2005). 31, 539–544. doi: 10.1111/j.1365-2214.2005.00548.x 16101649

[ref29] Miller A. L. Rosenblum K. L. Retzloff L. B. Lumeng J. C. (2016). Observed self-regulation is associated with weight in low-income toddlers. Appetite 105, 705–712. doi: 10.1016/j.appet.2016.07.007, PMID: 27397726 PMC4980170

[ref30] Must A. Strauss R. S. (1999). Risks and consequences of childhood and adolescent obesity. Int J Obes. 23, S2–S11. doi: 10.1038/sj.ijo.0800852 10340798

[ref31] OECD. Portugal, in education at a glance 2022: OECD indicators, Paris: OECD Publishing (2022).

[ref32] Ogilvie R. P. Patel S. R. (2017). The epidemiology of sleep and obesity. Sleep Health 3, 383–388. doi: 10.1016/j.sleh.2017.07.013, PMID: 28923198 PMC5714285

[ref33] Palmer F. B. Graff J. C. Jones T. L. Murphy L. E. Keisling B. L. Whitaker T. M.. (2018). Socio-demographic, maternal, and child indicators of socioemotional problems in 2-year-old children a cohort study. Medicine (United States) 97:e11468. doi: 10.1097/MD.0000000000011468 PMC6076199 29995806

[ref34] Pascoe J. M. Wood D. L. Duffee J. H. Kuo A. (2016). Mediators and adverse effects of child poverty in the United States. Pediatrics 137:e20160340 doi: 10.1542/peds.2016-0340, PMID: 26962239

[ref35] Sahoo K. Sahoo B. Choudhury A. Sofi N. Kumar R. Bhadoria A. (2015). Childhood obesity: causes and consequences. J. Family Med. Prim. Care. 4, 187–192. doi: 10.4103/2249-4863.154628, PMID: 25949965 PMC4408699

[ref36] Shriver L. H. Dollar J. M. Lawless M. Calkins S. D. Keane S. P. Shanahan L.. (2019). Longitudinal associations between emotion regulation and adiposity in late adolescence: indirect effects through eating behaviors. Nutrients 11. doi: 10.3390/nu11030517, PMID: 30823405 PMC6470565

[ref37] Sivertsen B. Harvey A. G. Reichborn-Kjennerud T. Torgersen L. Ystrom E. Hysing M. (2015). Later emotional and behavioral problems associated with sleep problems in toddlers: a longitudinal study. JAMA Pediatr. 169, 575–582. doi: 10.1001/jamapediatrics.2015.0187, PMID: 25867179

[ref38] Smith J. D. Fu E. Kobayashi M. A. (2020). Prevention and management of childhood obesity and its psychological and health comorbidities. Annu Rev. Clin. Psychol. 16, 351–378. doi: 10.1146/annurev-clinpsy-100219 32097572 PMC7259820

[ref39] Taveras E. M. Rifas-Shiman S. L. Bub K. L. Gillman M. W. Oken E. (2017). Prospective study of insufficient sleep and neurobehavioral functioning among school-age children. Acad. Pediatr. 17, 625–632. doi: 10.1016/j.acap.2017.02.001, PMID: 28189692 PMC5545152

[ref40] Touchette É. Petit D. Séguin J. R. Boivin M. Tremblay R. Montplaisir J. Y. (2007). Associations between sleep duration patterns and behavioral/cognitive functioning at school entry. Sleep 30, 1213–1219. doi: 10.1093/sleep/30.9.1213, PMID: 17910393 PMC1978413

[ref41] Tso W. Rao N. Jiang F. Li A. M. Lee S. FKW H.. (2016). Sleep duration and school readiness of Chinese preschool children. J. Pediatr. 169, 266–271. doi: 10.1016/j.jpeds.2015.10.064, PMID: 26608085

[ref42] Turnbull K. Reid G. J. Morton J. B. (2013). Behavioral sleep problems and their potential impact on developing executive function in children. Sleep 36, 1077–1084. doi: 10.5665/sleep.2814, PMID: 23814345 PMC3669074

[ref43] Valero-García A. V. Olmos-Soria M. Madrid-Garrido J. Martínez-Hernández I. Haycraft E. (2021). The role of regulation and emotional eating behaviour in the early development of obesity. Int. J. Environ. Res. Public Health 18. doi: 10.3390/ijerph182211884, PMID: 34831637 PMC8622852

[ref44] Vandekerckhove M. Cluydts R. (2010). The emotional brain and sleep: an intimate relationship. Sleep Med. Rev. 14, 219–226. doi: 10.1016/j.smrv.2010.01.002 20363166

[ref45] Vaughn B. E. Elmore-Staton L. Shin N. El-Sheikh M. (2015). Sleep as a support for social competence, peer relations, and cognitive functioning in preschool children. Behav. Sleep Med. 13, 92–106. doi: 10.1080/15402002.2013.845778, PMID: 24527839

[ref46] Vazsonyi A. T. Liu D. Blatny M. (2022). Longitudinal bidirectional effects between sleep quality and internalizing problems. J. Adolesc. 94, 448–461. doi: 10.1002/jad.12039, PMID: 35390199

[ref47] Werchan D. M. Gómez R. L. (2014). Wakefulness (not sleep) promotes generalization of word learning in 2.5-year-old children. Child Dev. 85, 429–436. doi: 10.1111/cdev.12149, PMID: 23962141

[ref48] WHO. Measuring a Child’s growth Department of Nutrition for health and development. Geneva: World Health Organization (2008).

[ref49] Williams K. E. Nicholson J. M. Walker S. Berthelsen D. (2016). Early childhood profiles of sleep problems and self-regulation predict later school adjustment. Br. J. Educ. Psychol. 86, 331–350. doi: 10.1111/bjep.12109, PMID: 26918668

[ref50] Zheng M. Rangan A. Olsen N. J. Heitmann B. L. (2021). Longitudinal association of nighttime sleep duration with emotional and behavioral problems in early childhood: results from the Danish healthy start study. Sleep 44, 5–8. doi: 10.1093/sleep/zsaa138, PMID: 32691048

