# Supplementary material for: Is the association between sleep and socio-emotional development mediated by weight in toddlers aged 12 to 36 months?
Source: Front Psychol. 2023 Nov 30;14:1190081. doi: 10.3389/fpsyg.2023.1190081 (PMC10731978; doi:10.3389/fpsyg.2023.1190081)
Supplement: Supplementary file 1 [file Data_Sheet_1.docx]

**Supplementary Information**

Table 1a. Linear regression models (β (95% Confidence Interval)) between sleep duration and anthropometric measures

|  | Weight (kg) | Weight for age z-score | Weight for age percentile | Height/length (cm) | Height for age z-score | Height for age percentile |
| --- | --- | --- | --- | --- | --- | --- |
| Nighttime sleep | -0.147 *  (-0.0002; -0.00001) | 0.003  (-0.00004; 0.00005) | -0.003  (-0.001; 0.001) | -0.071  (-0.001; 0.0002) | 0.191 *  (0.00003; 0.00013) | 0.171 *  (0.0004; 0.003) |
| Daytime sleep | -0.008  (-0.0001; 0.0001) | 0.045  (-0.00004; 0.00009) | 0.043  (-0.001; 0.002) | -0.040  (-0.001; 0.0003) | -0.010  (-0.0004; 0.0004) | -0.018  (-0.002; 0.002) |
| Total sleep | -0.119  (-0.0002; 0.000008) | 0.029  (-0.00003; 0.00005) | 0.027  (-0.001; 0.001) | -0.065  (-0.0004; 0.0001) | 0.186 *  (0.00002; 0.0001) | 0.136 *  (0.00002; 0.002) |

* p < 0.05

Table 1b. Linear regression models (β (95% Confidence Interval)) between sleep duration and anthropometric measures

|  | Waist (cm) | Waist z-score | BMI z-score | BMI percentile | Weight for height (z-score) | Weight for height (percentile) |
| --- | --- | --- | --- | --- | --- | --- |
| Nighttime sleep | -0.050  (-0.0002; 0.0001) | -0.050  (-0.00003; 0.00001) | -0.190 *  (-0.0001; -0.00002) | -0.171 *  (-0.003; -0.0004) | -0.140 *  (-0.0001; -0.000003) | -0.132  (-0.002; 0.000008) |
| Daytime sleep | 0.015  (-0.0002; 0.0002) | 0.015  (-0.00002; 0.00003) | 0.036  (-0.00005; 0.00009) | 0.044  (-0.001; 0.002) | 0.037  (-0.00005; 0.00009) | 0.040  (-0.001; 0.002) |
| Total sleep | -0.024  (-0.0002; 0.0001) | -0.024  (-0.00002; 0.00001) | -0.153 *  (-0.00009; -0.00001) | -0.124  (-0.002; 0.0001) | -0.109  (-0.00007; 0.000007) | -0.092  (-0.002; 0.0003) |

* p < 0.05

Table 2 – Linear regression models (β (95% Confidence Interval)) between anthropometric measures and socioemotional development

|  | SE development  scaled score | SE development  percentile |
| --- | --- | --- |
| Weight (kg) | -0.150 *  (-0.500; -0.051) | -0.140 *  (-3.925; -0.267) |
| Weight for age  z-score | 0.055  (-0.286; 0.741) | 0.050  (-2.506; 5.888) |
| Weight for age  percentile | 0.053  (-0.010; 0.025) | 0.052  (-0.083; 0.202) |
| Height/lenght (cm) | -0.224 *  (-0.190; -0.057) | -0.211 *  (-1.493; -0.402) |
| Height for age  z-score | -0.032  (-0.520; 0.309) | -0.026  (-4.103; 2.669) |
| Height for age  percentile | -0.041  (-0.023; 0.012) | -0.049  (-0.194; 0.084) |
| Waist (cm) | -0.093  (-0.277; 0.039) | -0.077  (-2.076; 0.491) |
| Waist z-score | -0.093  (-2.209; 0.310) | -0.077  (-16.546; 3.913) |
| BMI z-score | 0.095  (-0.106; 0.805) | 0.084  (-1.203; 6.248) |
| BMI percentile | 0.076  (-0.008; 0.032) | 0.076  (-0.063; 0.262) |
| Weight for height (z-score) | 0.114  (-0.035; 0.919) | 0.102  (-0.678; 7.132) |
| Weight for height (percentile) | 0.117  (-0.001; 0.040) | 0.111  (-0.018; 0.317) |

* p < 0.05

Table 3a – Linear regression models (β (95% Confidence Interval)) between sleep duration and socioemotional development with different adjustments (***p ≤ 0.05**)

| **Socio-emotional development** | | | | | |
| --- | --- | --- | --- | --- | --- |
|  | **Unadjusted Model** | **Adjusted Model^(1)^** | **Adjusted Model^(2)^** | **Adjusted Model^(3)^** | **Adjusted Model^(4)^** |
| **Nighttime sleep duration** | **0.223 (0.001; 0.004)*** | **0.190 (0.001; 0.004)*** | **0.159 (0.0001; 0.003)*** | **0.176 (0.001; 0.004)*** | **0.168 (0.0003; 0.003)*** |
| **Daytime sleep duration** | **-0.173 (-0.005; -0.001)*** | **-0.184 (-0.005; -0.001)*** | **-0.181 (-0.005; -0.001)*** | **-0.166 (-0.005; -0.001)*** | **-0.126 (-0.004; 0.0001)** |
| **Total sleep duration** | **0.062 (-0.001; 0.002)** | **0.024 (-0.001; 0.002)** | **-0.005 (-0.001; 0.001)** | **0.028 (-0.001; 0.002)** | **0.047 (-0.001; 0.002)** |

(1) Adjusted model for sex and age.

(2) Adjusted model for sex, age and total energy intake.

(3) Adjusted model for sex, age and mothers’ education.

(4) Full adjusted model for sex, age, mothers’ education, total energy intake, motor development (fine and gross), and BMI z-score.

Table 3b – Linear regression models (β (95% Confidence Interval)) between sleep duration and socioemotional development with different adjustments (***p ≤ 0.05**)

| **Socio-emotional development** | | | | | |
| --- | --- | --- | --- | --- | --- |
|  | **Adjusted Model^(5)^** | **Adjusted Model^(6)^** | **Adjusted Model^(7)^** | **Adjusted Model^(8)^** | **Adjusted Model^(9)^** |
| **Nighttime sleep duration** | **0.214 (0.001; 0.004)*** | **0.193 (0.001; 0.004)*** | **0.139 (-0.0001; 0.003)** | **0.199 (0.001; 0.004)*** | **0.175 (0.001; 0.004)*** |
| **Daytime sleep duration** | **-0.186 (-0.005; -0.001)*** | **-0.158 (-0.005; -0.001)*** | **-0.125 (-0.004; 0.0002)** | **-0.137 (-0.004; -0.0002)*** | **-0.140 (-0.004; -0.0003)*** |
| **Total sleep duration** | **0.040 (-0.001; 0.002)** | **0.042 (-0.001; 0.002)** | **0.020 (-0.001; 0.002)** | **0.065 (-0.001; 0.002)** | **0.044 (-0.001; 0.002)** |

(5) Adjusted model for sex, age and BMI z-score.

(6) Adjusted model for sex, age and motor development.

(7) Adjusted model for sex, age, mothers’ education, total energy intake and motor development.

(8) Adjusted model for sex, age, mothers’ education, BMI z-score and motor development.

(9) Adjusted model for sex, age, mothers’ education, and motor development.
